# Enhanced operation of female reproductive microphysiological system (MPS) for rapid mechanistic study

**DOI:** 10.1186/s40486-025-00246-0

**Published:** 2025-12-19

**Authors:** Po Yi Lam, Sungjin Kim, Haemin Jung, Rahul Cherukuri, Ramkumar Menon, Arum Han

**Affiliations:** 1 Department of Electrical and Computer Engineering, Texas A&M University, College Station, TX USA; 2 Division of Basic Science and Translational Research, Department of Obstetrics and Gynecology, The University of Texas Medical Branch at Galveston, Galveston, TX USA; 3 Department of Biomedical Engineering, Texas A&M University, College Station, TX USA; 4 Department of Chemical Engineering, Texas A&M University, College Station, TX USA

**Keywords:** Microphysiological system, Organ-on-chip, High throughput assay, Automation

## Abstract

Microphysiological systems (MPS) have shown their capabilities in mimicking in vivo-like structural and functional complexity and are seeing significant increase in their utilization in the field of drug discovery and toxicology. However, the major time-consuming steps in the fabrication, utilization, and analyses of MPS devices limit the throughput for broader adoption. Here, we advanced the previously developed two-chamber MPS model of the female reproductive tracts from a single unit chip to an array type chip that is compatible with multi-channel pipettor or automated liquid handling robot for rapid and more efficient operation. To enable this array model, a new microfabrication method was developed, incorporating a microplate holder, bonding guide plate, and soft lithography cassette to minimize device-to-device variation. To validate its compatibility with multi-channel pipettors in chemical toxicity testing, cadmium, a chemical previously shown to elicit cytotoxicity in the two-chamber feto-maternal interface MPS model, was utilized to demonstrate highly uniform cell loading (variance < 100 cells/mm^2^) and consistent dose-dependent cytotoxic response. Additionally, a liquid handling robotic system was also utilized, with no operational errors such as air bubble introduction (zero bubbles out of 100 devices) during cell/chemical loading process, and no unintended cytotoxic effects (> 97% viability). These results highlight that this automation-compatible array type MPS device can provide highly consistent cell culture performance and significantly reduced chip-to-chip and operation-to-operation variations, overcoming the limitations of typical MPS devices.

**Supplementary Information:**

The online version contains supplementary material available at 10.1186/s40486-025-00246-0.

## Introduction

There are growing demands for new alternative methodologies (NAMs) [1] in drug development and chemical toxicity testing models, as many commonly used animal models do not recapitulate human physiology, are costly, and also faces growing ethical concerns, while most in vitro models are too simplistic to faithfully mimic the structure and responses of in vivo organ systems [2]. Microphysiological system (MPS), also known as organ-on-chip (OOC) or tissue chip, has emerged as one of the most promising alternative models, bridging the gaps between 2D cell culture, ex vivo models, animal models, and clinical trials [3, 4]. Many microfluidics-based MPS models enable recreation of in vivo cellular characteristics and architecture of specific organ or tissue, several of these featuring sophisticated technologies such as integrated electrical/mechanical/biochemical sensing capabilities [5–7] and dynamic flow application to enable in vivo-level shear stress during culture [8, 9], to name a few. These advanced in vitro systems have been utilized for disease modeling [10–12], drug development [12–15], and chemical toxicity testing [16–18] and also potentially being coupled with artificial intelligence (AI) technology for better data analytics [19, 20]. The rise of interest in NAMs from researchers in academia and industry as well as regulators [1] for toxicity studies, pharmacokinetics and pharmacodynamics (PK/PD) studies, and adsorption, distribution, metabolism and excretion (ADME) profiling are predicted to have cost reduction of up to 26% in pharmaceutical research and development (R&D), saving more than $700 million upon adoption of MPS technologies [21].

Despite this growing interest, with market size estimated to reach over $6 billion by 2025 [22], the adoption of MPS models by end-users in the pharmaceutical and chemical industry has been often limited due to various challenges. General users prefer to use a standardized platform rather than complex architecture and operation protocols, and also expect high reliability, robustness, and reproducibility [23, 24]. Many MPS models from academia are still difficult to be adopted in general laboratories due to complexity in terms of both fabrication and operation [25]. Commonly used fabrication techniques include photolithography, soft lithography, 3D printing, laser cutting, and injection molding require special equipment that is not often available in most biological laboratories and often times require a well-established cleanroom facility for fabrication [26]. For operation of MPS devices, liquid loading and sampling can be tedious while some platforms require continuous media flow controlled by external pumping systems, which further increase the workload or complexity of operation [25, 27–29]. Microscopy imaging is one of the key endpoint readouts in MPS devices; however, having to take large number of images drastically increases not only the image acquisition time but also analyses time [30, 31]. On the other hand, MPS devices from industry typically have relatively simple structure and increased usability [32, 33]. Furthermore, industry-scale manufacturing processes allow mass production of MPS devices necessary for higher throughput screening [34]. Thus, although MPS models from industry are sometimes oversimplified, they have been more widely adopted and utilized. However, they still remain quite expensive [21]. Most importantly, regardless of the MPS models, many of them still do not have the necessary throughput for broad usage in drug and chemical testing applications. First, microfabricating MPS devices still remain a relatively low throughput process, especially polydimethyl siloxane (PDMS)-based MPS devices, which are still very commonly utilized due to its ease of fabrication despite several limitations such as molecular adsorption/absorption [35–37]. This is in part due to difficulties in fabricating and assembling injection-molded plastic MPS devices as well as low oxygen permeability limitations [38, 39]. A method that allows easier microfabrication of PDMS-based MPS devices can be beneficial, such as eliminating the process of biopsy punching to create inlets and outlets for the culture chamber. Reported strategies include directly molding from 3D-printed masters with chamber structures integrated with pillars [40] or inserting needles before PDMS curing [41] to define such ports. However, these approaches are generally limited to simple platforms with feature sizes in the millimeter to submillimeter range. Second, cell loading into MPS devices is a time-consuming and labor-intensive steps, especially for multi-cellular co-culture MPS devices. Being able to load the same cell types into multiple MPS devices can significantly reduce this labor. Finally, culture media exchange, reagent loading, and effluent sampling are many of the time-consuming and manual steps, especially when time-course experiments are desired, which can again benefit from a simpler operating step.

Most female reproductive studies have been conducted with animal models and simplified 2D cell culture models. However, these conventional platforms cannot recapitulate the architectural complexity and responses observed from human organ systems [42]. Several female reproductive MPS models have been established over the past decade [42]. The first placenta-on-a-chip was proposed in 2016, which consisted of two cell culture compartments separated by a porous membrane [43]. In the subsequent year a modular multi-organ system to simulate the female reproductive tract and the endocrine feedback loops between organs have been developed, which successfully modeled the 28-day menstrual cycle observed in female reproductive system [44]. We have previously developed various MPS models that can recreate female reproductive tracts, including fetal membrane [10, 11], placental barrier [45], 2nd trimester placental tissue [46], low uterine tissue representing cervix and vaginal area [47, 48], and multi-organs [49]. Especially, the well-established two-chamber amnion-on-a-chip device has been utilized for studies of cellular transition observation and disease modeling [50], investigating the effect of dynamic fluid flow [8], monitoring immune cell migration [51], normal and pathological cellular remodeling of the cervix [52], and preterm birth (PTB)-related fetal neuroinflammation [53]. All of these established systems utilize horizontal co-culture approach with cell-specific culture chambers interconnected with arrays of microchannels. The microchannels were designed to avoid direct mixture of media and cells immediately after loading, and enable diffusion of small molecules between the chambers. Each culture chamber was connected to reservoirs, which allow supply of additional media, chemical treatment from specific cell layers, and collection of effluent from each cell layer. Despite this diverse range of studies, this two-chamber MPS model requires many individual and manual operation steps during fabrication and experiments, thus still limiting.

**Fig. 1:**
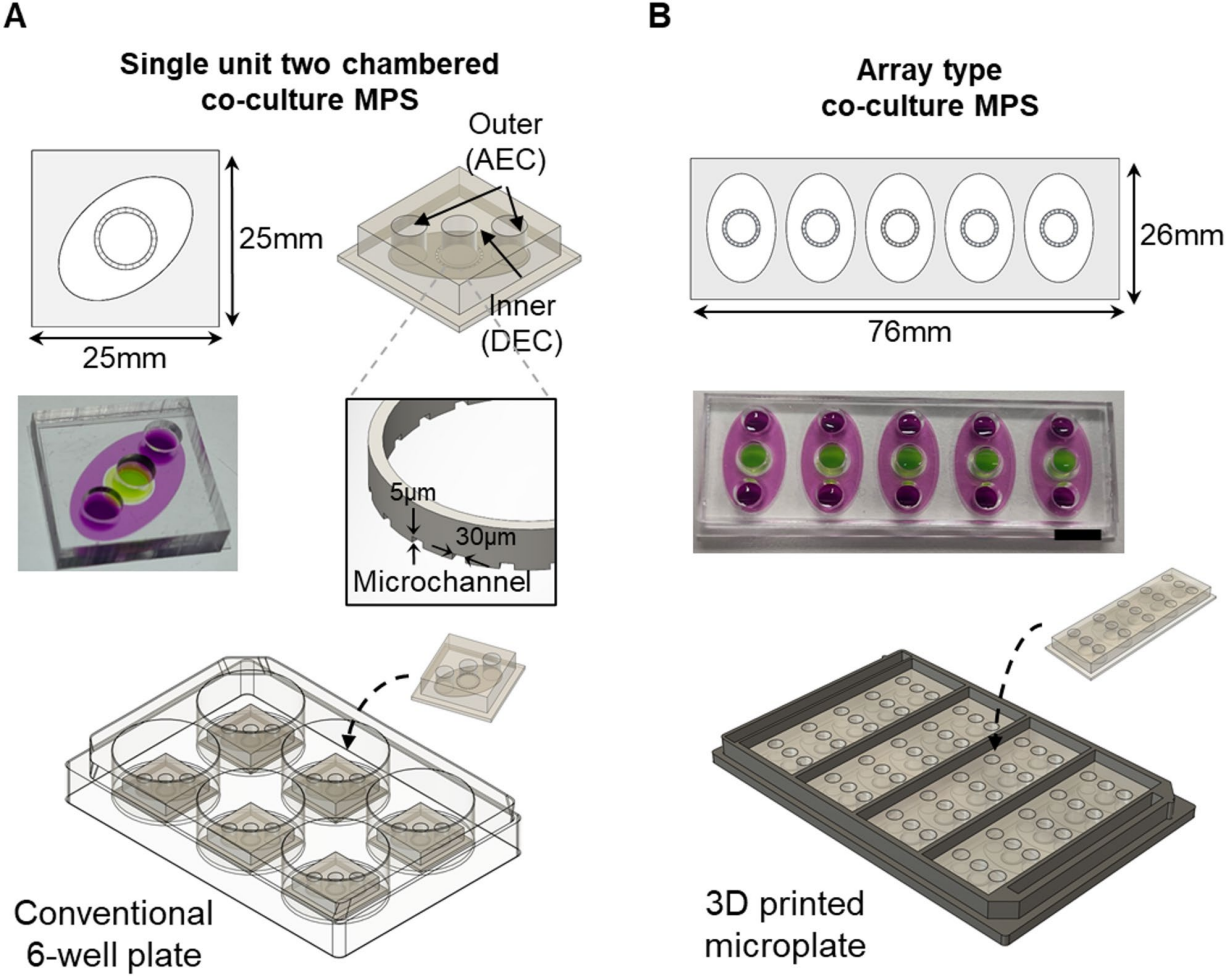
Design of a single unit type and array type two-chamber co-culture MPS model. **A** Schematic drawings and image of a single unit MPS device within a well of a 6-well plate. **B** Schematic drawings and image of the arrayed two-chamber MPS device integrated into a customized 3D-printed microplate (96-well plate size). Scale bar = 1 cm

In this paper, we modified this two-chamber co-culture MPS model from single unit into an array format to enhance its fabrication and operational efficiency (Fig. 1). The arrayed model has five replicates on a single cover slip (20 × 70 mm) with uniform distances between each device. The utility of this array type MPS model was evaluated by demonstrating two types of operation methods, (1) manual multi-channel pipette operation and (2) semi-automatic liquid handling using a liquid handling robotic system, for cell loading as well as culture media / reagent loading and sampling. The device and operation were evaluated through basic cytotoxicity assay as well as CaCl_2_ (Cd) exposure study that mimics our previously published study [54]. For microfabricating the array type MPS device, 3D printing technology combined with a newly developed soft lithography cassette, bonding guide plate, and microplate holder were utilized to increase the fabrication efficiency and minimize defects. Overall, the developed platform provides an avenue towards how to convert manual operation low-throughput MPS devices into higher-throughput MPS devices that are easier to fabricate and operate.

## Methods

### Fabrication

#### MPS array device master mold

The two-chamber MPS array model (5 device in a row x 4 rows = 20 device on five 75 mm x 26 mm glass substrates) was fabricated using a two-step photolithography and soft lithography process, as previously described [8, 11, 51, 54]. In brief, the master mold was created on a 4-inch silicon substrate using two layers of photosensitive epoxy (SU-8; MicroChem, Westborough, USA), each with a different thickness. The first layer, forming the 5-µm-high microchannels, was fabricated by spin-coating SU-8 3005 at 3400 rpm for 45 s, followed by soft baking at 95 °C for 2 min. The layer was then UV-exposed through a photomask, post-exposure baked at 65 °C for 1 min, and then at 95 °C for 2 min. The second layer, creating the 500 μm thick cell culture chambers, was fabricated by spin-coating SU-8 2100 at 1,000 rpm for 45 s, soft baked at 95 °C for 1 h, UV-exposed through a second photomask, and post-exposure baked at 65 °C for 5 min, followed by another bake at 95 °C for 15 min. To ensure proper SU-8 structure formation, the mold was immersed in edge-bead removal solution (MICROPOSIT EBR-10 A) for 15 min, rinsed with isopropyl alcohol (IPA) and deionized (DI) water, and then coated with (tridecafluoro-1,1,2,2-tetrahydrooctyl) trichlorosilane (United Chemical Technologies, Bristol, PA, USA) to facilitate the release of the polydimethylsiloxane (PDMS) layer.

#### PDMS array device fabrication cassette

**Fig. 2:**
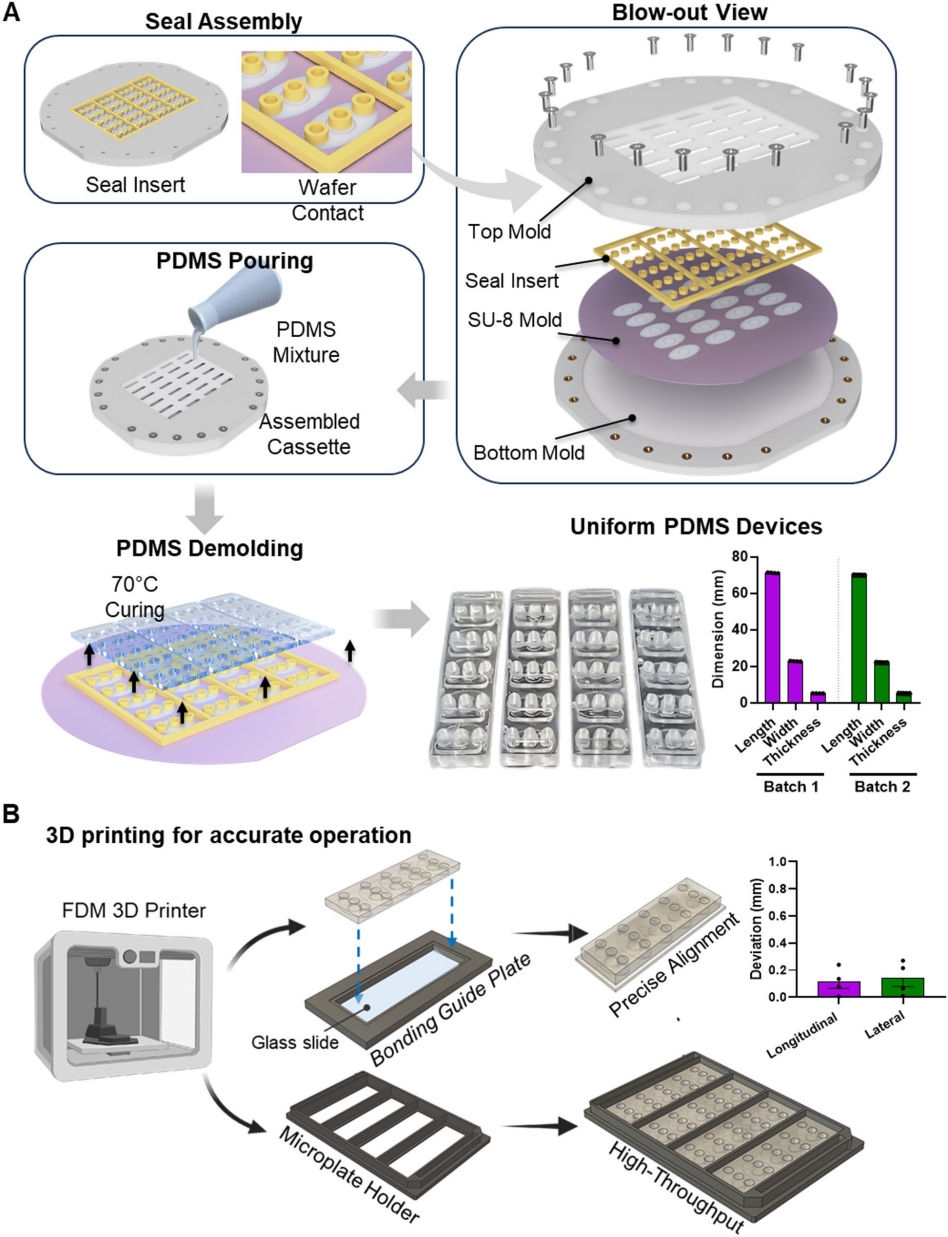
Schematic diagram of the array device fabrication step, followed by mounting onto a microplate holder for ease of automation. **A** The SU8 master mold wafer was assembled with a 3D-printed elastic seal layer forming the inverse replica of the culture chambers to eliminate the need for manual punching. This layer was then sandwiched between a top and bottom mold, where PDMS was poured through the top mold. Blow-out view displays the layers within the assembled cassette. **B** PDMS-based MPS array was mounted onto a 3D-printed bonding guide for precise alignment of the PDMS replicates to glass substrates, followed by mounting the assembly into a 3D-printed microplate holder to maintain position accuracy of the device to enable cell/media loading using a multi-channel pipettor or a liquid handling robot

The SU-8 master mold, as described in the previous section, was placed into a 3D-printed PDMS molding cassette, which is designed to ease the PDMS fabrication process (Fig. 2A). This cassette design eliminates the need for the typical PDMS reservoir punching and device cutting procedures needed in conventional soft lithography process, ensuring that the final device has minimum device-to-device variability after microfabrication. This standardization is essential to guarantee consistent device fabrication and performance, particularly when the fabricated devices are used in conjunction with a robotic liquid handler. The PDMS molding cassette was designed using Fusion 360 software (Autodesk, CA, USA) and printed with a fused deposition modeling (FDM) 3D printer (X1E, Bambu Labs, Austin, USA) utilizing ABS filament (ABS white 40100 filament, Bambu Labs, Austin, USA). The cassette was designed with a top mold to secure the seal insert and a bottom part to hold the SU-8 patterned silicon wafer. When the parts were assembled and the screws tightened, the top and bottom molds applied compression, ensuring full contact between the seal insert and the SU-8 patterned silicon wafer. This prevents liquid PDMS from leaking through the seal insert. The seal insert features pillar and wall structures, which allow the chamber inlets and the edges of each array device to be molded directly during the soft lithography process, eliminating the need for manual biopsy punching and PDMS cutting. Following the 3D printing process, the cassette was subject to surface smoothing via acetone vapor treatment (ambient temperature, 30 min) to enhance the surface smoothness. The sealing interface of the cassette, placed on top of the SU-8 master mold, was printed in an elastic resin (Silicon 40 A resin) using a 3D resign printer (Formlabs, MA, USA). This elastic structure enables forming a tight seal between the mold and the cassette. This elastic sealing layer was secured to the cassette by aligning to the designed pillar structures on the top mold. The complete cassette was then coated with trichlorosilane to facilitate the release of the PDMS replicates.

Once the SU-8 mold was securely placed within the cassette, a 10:1 mixture of PDMS prepolymer (Sylgard 184; DowDuPont, Midland, USA) was poured into the cassette. The cassette was then placed in a vacuum chamber for 30 min to eliminate air bubbles. Subsequently, the cassette was cured in an oven at 75 °C for 60 min. After curing, the PDMS was carefully removed from the cassette, subjected to oxygen plasma treatment (Harrick Plasma, Ithaca, USA) for 120 s, and then bonded to a glass substrate (76 mm x 26 mm size) using the bonding guide plate for precise alignment (Fig. 2B). Thickness and size of devices were measured by a digital caliper (NEIKO) to confirm the dimension accuracy and consistency among replicates.

#### PDMS array device plate holder

The final bonded PDMS-glass devices were integrated into a custom-built microplate holder to facilitate their use within an automated liquid handler system. To minimize evaporation during incubation, two reservoirs containing PBS were incorporated in the microplate design. This plate holder was designed using the same software as the PDMS cassette and 3D printed using PA6-GF filament (PA6-GF, Bambu Labs, Austin, USA) with the same FDM X1E 3D printer. To enhance the mechanical property of the printed plate and enable autoclaving for sterilization, the plate was annealed at 100 °C for 12 h before use.

### Automatic operation of the MPS array device

The OT-2 automated liquid handling robot (Opentrons, NY, USA) was utilized for automating the device washing and cell loading processes. A custom JSON file was generated to specify the individual positions of each media well within the MPS device, enabling precise programming of the robot to interface with the devices placed within the microplate. The Opentron software was employed to manage the interaction between the individual microplates and the corresponding liquids.

### Cell culture

Immortalized cell lines, human decidua cells (hFM-DEC; DEC) and human amnion epithelial cells (hFM-AEC; AEC), utilized in this study were derived from human term fetal membranes with protocol reported in our previous publications [10, 45, 54, 55]. AEC culture was maintained in KSFM supplemented with epidermal growth factor (0.1 ng/mL), bovine pituitary extract (30 µg/mL), CaCl_2_ (0.4 mM), and primocin (0.5 mg/mL). DEC was cultured in DMEM/F12 supplemented with 10% FBS, 1% penicillin/streptomycin, and 1% amphotericin B. Both cell lines were maintained in a 37 °C incubator with 5% CO_2_. Media was replaced every two days and cells were harvested when it reached 80–90% confluency. All experiments were performed within 30 cell passages.

### Cell seeding and cadmium treatment

To sterilize the device, cell culture chambers and reservoirs were filled with 70% ethanol for 15 min followed by washing with complete DMEM/F12 media twice. Multichannel pipettes and automated liquid handling robots could also be utilized for ethanol and media loading in this cleaning process when devices are placed on the 3D-printed microplate holder. Then, AEC and DEC were harvested by trypsinization, resuspended in their cell-specific media, and loaded into the corresponding cell compartments (250,000 AECs and 30,000 DECs to each chamber) using multichannel pipettes and automated liquid handling robots with devices secured with the 3D-printed microplate holder. Devices along with the microplate holder were then incubated at 37 °C with 5% CO_2_ for 1 h to allow initial cell attachment to the devices. The reservoirs were filled with cell-specific media for overnight incubation before chemical treatment.

Cadmium chloride (CdCl_2_; Cd) was selected as the model environmental toxicant to be tested on the developed fetal membrane arrayed MPS device, since the effect of cadmium on feto-maternal interface was previously reported using our previous single-unit MPS device [54]. Cd (40 mM stock), dissolved in dimethyl sulfoxide (DMSO), was diluted in DMEM/F12 complete media to obtain 1, 10, and 100 µM of Cd. DMSO was supplemented accordingly to ensure all treated conditions contained 0.5% DMSO as the vehicle. Media was removed from all reservoirs without inducing cell detachment from the cell culture compartments, and the conditioned media (100 µl) was then added to the inner reservoirs and 50 µl of fresh AEC media was added to each outer reservoir to establish gravity-driven diffusion of Cd across the cell culture compartments, where the diffusion profile has been reported by monitoring the diffusion of FITC dye [50]. A multi-channel pipette was utilized to reduce the operation time and minimize handling errors. The devices were then incubated at 37 °C with 5% CO_2_ for 48 h for endpoint analysis. In subsequent experiments, a robotic liquid handler was utilized (see Sect. "Automatic operation of the MPS array device" for details).

### Cell viability

To evaluate responses from AECs and DECs after Cd exposure, lactate dehydrogenase (LDH) assay (Cat. No. 11644793001, Roche, Switzerland) and apoptosis/necrosis staining (Cat. No. V13241, Invitrogen, CA, USA) were performed. Effluent (50 µl) from each cell culture compartment was collected and LDH assay performed according to the vendor protocol. Cell-free culture media was used as the background control. To collect the high control (maximum amount of LDH from each culture), 50 µl of 10% Triton X-100 solution was loaded into each culture compartment to collect the cell lysate after 30 min of incubation. The cell lysates were diluted 10 times with the cell-specific culture media before mixing with the fresh reaction mixture. Samples were incubated for 30 min at room temperature under a dark environment and absorbance was measured at 490 nm using a microplate reader (SYNERGY H1, BioTek, VT, USA).

To assess the viability and distribution of cells seeded by the OT-2 automated liquid handling robot, LIVE/DEAD Viability/Cytotoxicity kit (Cat. No. L3224, Invitrogen, CA, USA) was used to stain cells after overnight incubation. Cells were incubated with calcein-AM (1:500 dilution), ethidium homodimer-1 (EthD-1, 1:2000 dilution), and H33342 (1:1000 dilution) in 1X phosphate buffered saline for 15 min. Fluorescent microscopy of stained cells in devices was performed using a fluorescent microscope (BZ-X810, Keyence, IL, USA) and analyzed by ImageJ software to count cells from three different regions or the entire device.

### Statistical analysis

All experiments were conducted with more than five replicates. The Prism 8 software (GraphPad Software, La Jolla, CA, USA) was utilized for graph plotting, with standard error of mean displayed as error bars in the plots. Statistical analysis was performed using the one-way ANOVA test, with *p* < 0.05 considered as significant.

## Results

### Array type two-chamber MPS model design and fabrication method development

The original two-chamber co-culture MPS model was designed as a 25 mm x 25 mm device that fits within a well of a 6-well plate (Fig. 1A). Although this model has been utilized for multiple studies [50, 56], it is not an ideal platform to achieve reasonable throughput as these devices have to be individually fabricated and handled for operation. To overcome these challenges and increase the throughput, a critical aspect for these MPS devices to be utilized more broadly and routinely, an arrayed two-chamber MPS model having 5 individual two-chamber MPS devices on a 76 mm x 26 mm footprint has been designed (Fig. 1B). A custom 3D-printed microplate (Supplementary Fig. 1A) having the same footprint as a well plate (127 mm x 85 mm) divided into four equal compartments to hold four arrayed MPS devices each (20 MPS devices in total) was developed. To capture microscopic images of cells within the devices, the bottom of the 3D-printed microplate was completely opened to enable imaging directly through the glass substrate without affecting the working distance (WD) of the objective lenses (4X objective with WD = 16.5 mm; 10X objective with WD = 14.5 mm).

To improve the throughput and easiness of the fabrication process, a soft lithography molding cassette (Supplementary Fig. 1B) was fabricated using a FDM 3D printer. The cassette is composed of a bottom mold with a footprint to secure a 6-inch diameter wafer and a top mold with pillars (dimension: 5 mm) holding the seal insert in contact with the SU-8 mold to create cell loading inlets/outlets directly during the PDMS curing process (Fig. 2A). To utilize the molding cassette, seal insert was first assembled onto the top mold that acts as a mold for the inlets and outlets. Then, the SU-8 mold with the cell culture chamber patterns was placed within the bottom mold. After aligning the top mold with seals to the bottom mold, the entire cassette was assembled with screws. PDMS mixture was then poured through the openings on the top mold and degassed to ensure no bubbles were trapped inside the cassette before baking. The cassette was disassembled after baking and four of the arrayed MPS devices were released from the mold. Devices fabricated with this new method resulted in inlets/outlets directly formed after the demolding process as shown in Supplementary Fig. 1C (all inlets/outlets formed within their corresponding cell culture chambers; inlet diameter = 3.96 ± 0.05 mm) and uniform device size (length = 71.20 ± 0.094 mm; width = 22.85 ± 0.061 mm; thickness = 5.18 ± 0.015 mm). Compared with the holes obtained by biopsy punch, the molded inlets/outlets also exhibited smooth edges, and no PDMS residue was observed from inlets/outlets, indicating that a leak-tight seal formed during the PDMS incubation.

A bonding guide plate was designed for precise alignment when bonding the demolded arrayed PDMS devices to a glass slide to minimize variations in orientation and spacing from the edge of the glass slide (Fig. 2B). After oxygen plasma treatment of the PDMS devices and glass substrate, the glass substrate was first placed to the slide trench within the bonding guide plate, and the PDMS replicate was then aligned and placed on the glass slide for bonding. This approach ensured precise alignment of the PDMS device to the center of the glass slide (longitudinal deviation = 0.115 ± 0.043 mm; lateral deviation = 0.14 ± 0.053 mm), resulting in minimal variation on device coordinates, an important aspect of MPS device operation using multi-channel pipettors or liquid handling robotic systems.

### Multi-channel pipette operation

#### Uniform distribution of cells during cell loading

**Fig. 3:**
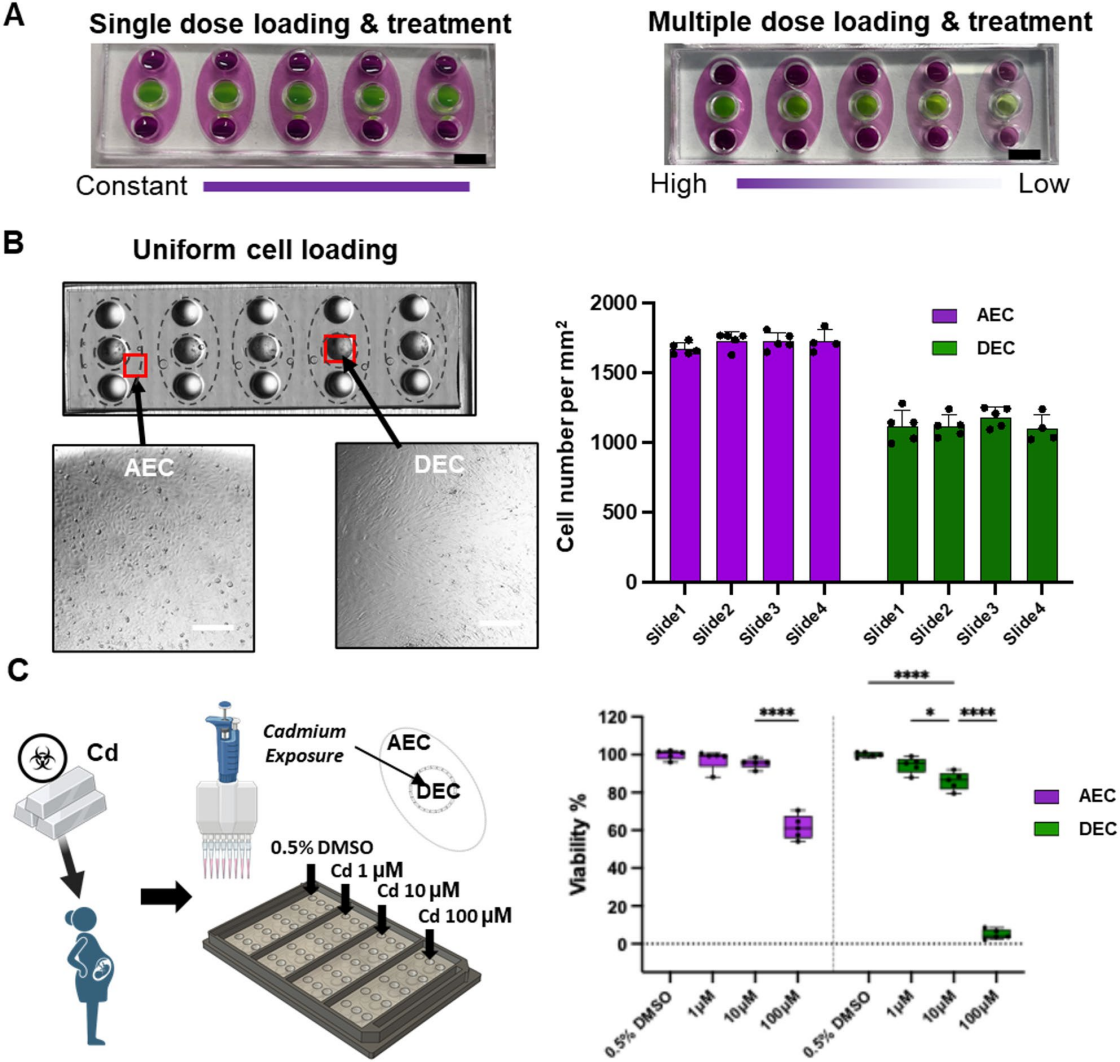
Performance of multi-channel pipette operation using the array type MPS model. **A** Array type MPS model enables easier and quicker single- and multiple-dose chemical loading into the MPS devices. Scale bar = 1 cm. **B** Uniform number of cells were loaded into the arrayed MPS device (*n* = 5). Scale bar = 50 μm. **C** Assessing effect of Cd exposure at 3 different concentrations compared to vehicle control (media with 0.5% DMSO) based on LDH-based cytotoxicity assay (*n* = 5)

Cell loading into MPS devices is the first time-consuming and labor-intensive step, as minimizing bubble formation while achieving uniform cell loading is critical for successful operation of the MPS devices. The original MPS design with a single device placed within a well of a 6-well plate does not allow operation with multi-channel pipettes due to the random device-to-device distances and orientation of cell loading inlets within the MPS devices. In addition, when handling large number of devices, rapid loading of cells into all devices is important to prevent decrease in cell viability due to the long cell loading time. First, to check uniform media loading into the outside and inside cell culture chambers, color dye at the same concentration (Fig. 3A left) as well as serial dilution (Fig. 3A right) was loaded into each of the devices using a multi-channel pipettor without creating bubbles. This was accomplished with 3 media loading steps into the 5-array device compared to requiring 15 loading steps for 5 single-unit devices. When operating several tens of devices, this reduction in steps becomes significant. Next, to assess cell loading uniformity, both AECs and DECs were loaded into their respective cell culture chambers and their distribution within the chambers inspected using brightfield (BF) microscopy (Fig. 3B), where uniform number of cells in each device was observed. The total number of cells loaded into each chamber were also analyzed to evaluate the consistency of device-to-device cell numbers within the 5-device array as well as between different arrays (Fig. 3B). Results show consistent AEC and DEC numbers when cells were seeded into all 5 devices within the same arrayed device with a single cell loading operation (AEC number = 1713.1 ± 47.3 cells/mm^2^; DEC number = 1131.2 ± 87.0 cells/mm^2^), while no significant differences were observed when compared to different arrays.

#### Consistent cytotoxicity responses from environmental toxicant exposure

To confirm the reproducibility of cytotoxicity responses resulting from consistent cell density across replicates, Cd was applied to the maternal DEC compartment at 1, 10, 100 µM concentrations using a multi-channel pipettor, expecting propagation of Cd to the fetal AEC compartment through the microchannel array by hydrostatic pressure [50]. After 48 h, effluents were collected from each chamber to measure LDH production from cells to evaluate the cytotoxicity by Cd exposure. As expected, and as previously reported in a single-unit MPS device study [54], dose-dependent cytotoxicity could be observed from both the DEC and AEC compartments (Fig. 3C). As DECs were directly exposed to Cd, significant cell death was induced starting from 10 µM Cd exposure (*p = 0.0146), while 100 µM Cd exposure resulted in >90% cell death (****p < 0.0001). Significant cytotoxic effect on AECs at the fetal side was only observed from indirect 100 µM Cd exposure (***p < 0.0001). This result shows that with consistent number of cells between devices and minimized pipetting errors, consistent cytotoxicity responses amongst the replicates could be observed from this Cd environmental toxicant exposure study.

### Automatic operation

**Fig. 4:**
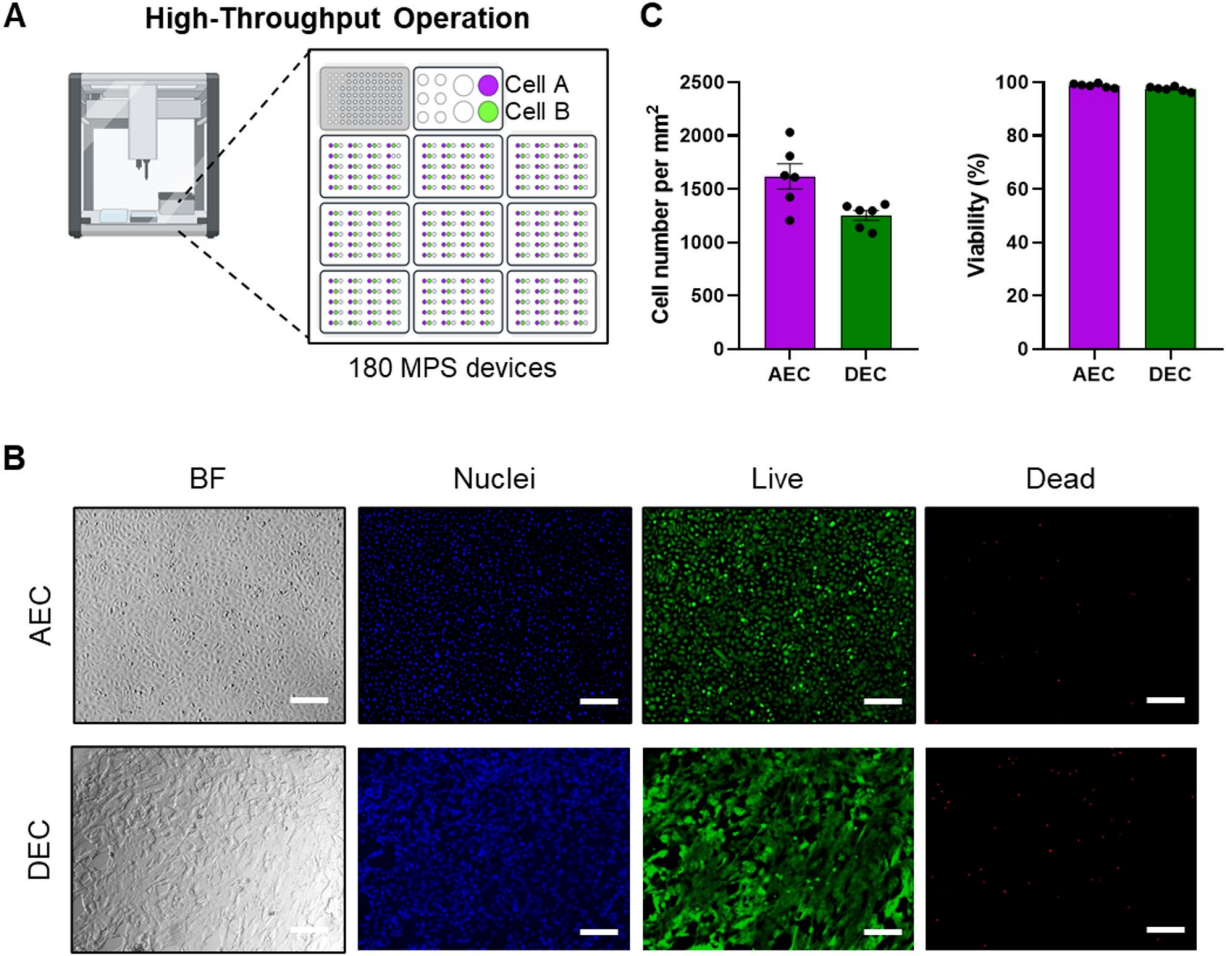
Performance of automatic operation using the array type MPS model. **A** Overview of the automatic operation. **B** Live/dead cell fluorescent microscopy. **C.** Statistical analysis of the automated cell loading performance in terms of cell number and viability among replicates (*N* = 5). Scale bar = 200 μm

With the modified soft lithography process using the 3D-printed cassette for device manufacturing, each cell loading inlets are molded at the same time, minimizing the variation in device-to-device and inlet-to-inlet locations. This enabled the usage of automatic liquid handling systems for cell seeding, media replenishment, and chemical treatment by programming the movement of the robotic pipettor. The OT-2 automated liquid handling system can accommodate nine well plates (180 arrayed-MPS replicates in total) as shown in Fig. 4A. Color dye loading was first performed (Supplementary Fig. 2) to demonstrate the capability of the automated operation for both the outer and inner cell culture chambers. Liquid placed in “Cell A” tube was loaded into the outer chambers through inlets labeled with purple color while that from “Cell B” tube was added to the inner chambers labeled with green color. As a result, the automatic liquid handling system was able to load reagents into each device efficiently without inducing air bubbles within the cell culture chambers.

To confirm the feasibility of utilizing the OT-2 automated liquid handling robot for cell loading, AEC and DEC cell suspensions were prepared in 50 ml conical tubes us Cell A and Cell B, respectively, for cell loading to the arrayed models. Cell distribution within the cell culture chambers and viability were monitored after 48 h-incubation using live/dead fluorescent staining (Fig. 4B). Consistent cell numbers and well-maintained viability resulted from both AECs (density = 1618.2 ± 107.6 cells/mm^2^, viability = 98.82 ± 0.28%) and DECs (density = 1252.8 ± 41.8 cells/mm^2^, viability = 97.52 ± 0.33%), indicating that the automated liquid handling robot could ensure consistency in cell seeding density between devices at a significantly reduced operation time (1 h vs. 10 min for 40 devices) without impacting the cell viability.

## Discussion

Conventional 2D and 3D culture platforms, such as well plates and organoids, remain most commonly used due to their simplicity, reproducibility, and cost effectiveness [57, 58]. However, these platforms lack critical in vivo-like features such as multicellular structures, intercellular signaling, and ECM integration, limiting their physiological relevance [58–60]. Microfluidic MPS technologies address these gaps by recreating tissue architecture and functions, making them valuable tools for PK/PD modeling, disease studies, drug testing, and toxicological testing [13, 23]. Despite their promises, broader adoption has been limited by complex fabrication and operation as well as time-consuming steps, especially for high-throughput use, highlighting a trade-off between usability, scalability, and biological fidelity [21, 24, 25]. In this study, we present a platform that advances the previously established single-unit two-chamber feto-maternal interface MPS [54] into an arrayed format. The original single-unit model, which fits in a well of a 6-well plate, has a larger footprint and relies on manual fabrication and operation, which are time-consuming and prone to human error. These limitations compromise production quality and hinder the reproducibility of test/study outcomes, significantly restricting the platform’s usability for automation and its potential for higher-throughput screening, an essential requirement in pharmaceutical toxicological research [61–64]. In contrast, the developed arrayed MPS model offers substantial improvements in both fabrication and operation, as summarized in Table 1. By integrating a 3D-printed cassette into the PDMS soft lithography fabrication process, we minimized fabrication errors typically associated with manual cutting, biopsy punching of inlets/outlets, and inconsistencies in device thickness due to uneven PDMS volumes or mold leveling during curing. This design enabled the direct formation of standardized inlets at fixed positions during molding, thereby reducing fabrication time and improving device uniformity. The in situ formation of inlet and outlet structures also allowed batch production of 20 spatially aligned replicates per mold, significantly reducing device-to-device dimensional variation. Uniform inlet diameters (deviation = 0.05 mm) and consistent device sizes (dimension deviation < 0.1 mm) achieved through this process contributed to consistent fluidic resistance and diffusion profiles across the array. Maintaining a uniform inlet size is particularly important for ensuring consistent diffusion profile among replicates as variations in inlets diameter will alter the media level, which will then directly affect the hydrostatic pressure-driven flow. These factors are critical for ensuring reproducible test outcomes using MPS platforms [13].

**Table 1:** Comparison between the original single-unit MPS device and the developed arrayed MPS in terms of fabrication and operation process

	Single unit	Arrayed MPS (this work)
Fabrication quality	• Human error from cutting and biopsy punching of inlet/outlet	• Minimized manual operation • Uniform thickness and size • Standardized inlet/outlet positions
Fabrication time & cost	• Time-consuming inlet/outlet biopsy punching process	• No need for device cutting and biopsy punching
Operation	• Single device loading • Manual loading • Slow • Low throughput	• Compatible with multi-channel pipette and automatic liquid pipettors • Capable of multiple dose treatment • Higher throughput
Operation cost	• High labor cost • Time consuming	• Reduced time and labor cost

Operational efficiency was also further enhanced through compatibility with both multi-channel pipettors and automated liquid handling systems. This enabled reliable cell seeding, media replenishment, and reagent delivery without the introduction of air bubbles into the cell culture chambers. Compared to the single-unit device, which requires extensive manual operation, the arrayed system allows significantly more efficient and higher-throughput operation. Multi-channel pipetting provides a practical and accessible alternative to robotic automation, reducing hands-on time and operational costs, particularly beneficial for small- to medium-scale experiments. Additionally, the precise alignment of inlet and outlet positions across replicates of the arrayed MPS enabled the use of programmable robotic liquid handlers. This scalability supports full automation, making the platform highly suitable for industrial applications such as drug/toxicant library screening [21, 65].

Our results demonstrate that the platform supports high cell viability (>97% live cells up to 48 h) and low inter-device variability in cell density (variance < 100 cells/mm²), enabling uniform exposure to treatment conditions. This was validated by cadmium chloride cytotoxicity assays, which produced dose-dependent responses consistent with previously reported results that used single-unit MPS devices [54]. These findings demonstrated the robustness and scalability of the developed arrayed MPS model, making it a promising alternative for toxicological screening, drug screening, disease modeling, and mechanistic investigations.

In conclusion, by addressing current limitations in fabrication complexity, operational inefficiency, and biological variability in MPS devices, the developed arrayed model enhances reproducibility and throughput of experiment. This MPS device is both scalable and automation-compatible while maintaining high cell viability and uniform biological responses. This advanced platform can serve as a robust and versatile tool for toxicological screening, drug screening, disease modeling, and mechanistic studies, potentially applicable to industrial automation workflows beyond academic research. As the demand for standardized and scalable MPS continues to grow, this platform offers a practical and effective solution for converting a pre-existing single-unit MPS device into arrayed format, promoting the adoption of organ-on-chip technologies in both pharmaceutical and regulatory contexts.

## Supplementary Information

Below is the link to the electronic supplementary material.

Supplementary Material 1.

## Data Availability

The data that support the findings of this study are available on request from the corresponding author.
