# Supplementary material for: Enhanced operation of female reproductive microphysiological system (MPS) for rapid mechanistic study
Source: Micro Nano Syst Lett. 2025 Dec 19;13(1):26. doi: 10.1186/s40486-025-00246-0 (PMC12755145; doi:10.1186/s40486-025-00246-0)
Supplement: Supplementary file 1 — Supplementary Material 1. [file 40486_2025_246_MOESM1_ESM.docx]

**Enhanced Operation of Female Reproductive Microphysiological System (MPS) for Rapid Mechanistic Study**

Po Yi Lam^1*^, Sungjin Kim^1*^, Haemin Jung^1^, Rahul Cherukuri^1^, Ivan Rusyn^2^, Ramkumar Menon^3^, and Arum Han^1,4,5**^

^1^ Department of Electrical and Computer Engineering, Texas A&M University, College Station, Texas, USA

^2^ Department of Veterinary Integrative Biosciences, College of Veterinary Medicine and Biomedical
 Sciences, Texas A&M University, College Station, Texas, USA

^3^ Division of Basic Science and Translational Research, Department of Obstetrics and Gynecology, The

University of Texas Medical Branch at Galveston, Galveston, Texas, USA

^4^ Department of Biomedical Engineering, Texas A&M University, College Station, Texas, USA.

^5^ Department of Chemical Engineering, Texas A&M University, College Station, Texas, USA.

*Both authors contributed equally to this manuscript

**** Corresponding author:**

Arum Han, PhD

Professor, Department of Electrical and Computer Engineering

Department of Biomedical Engineering

Department of Chemical Engineering

Texas A&M University

E-mail: [arum.han@ece.tamu.edu](mailto:arum.han@ece.tamu.edu)

**Supplementary Figure**

**Supplementary Figure 1.** 2D drawing of the **(A)** microplate holder and the **(B)** PDMS array device fabrication cassette consisting of (i) seal insert, (ii) SU-8 patterned wafer, (iii) top mold with pillar structure for inlets/outlets molding, and (iv) bottom mold to secure the wafer. **(C)** BF images of PDMS replica fabricated by the 3D-printed cassette. Scale bar = 4mm.

**Supplementary Figure 2.** **(A)** Images demonstrating the operation of automatic liquid handler for loading different color dye into the assigned cell culture chambers. **(B)** A representative image of the device after cell loading, with four zoomed-in images from specific regions. Scale bar = 1 mm.
